# Clinical characteristics associated with the onset of delirium among long-term nursing home residents

**DOI:** 10.1186/s12877-018-0733-3

**Published:** 2018-02-02

**Authors:** Evelyn Ning Man Cheung, Sophiya Benjamin, George Heckman, Joanne Man-Wai Ho, Linda Lee, Samir K. Sinha, Andrew P. Costa

**Affiliations:** 1 Department of Medicine, McMaster University, IHB/HSC-McMaster 3016, Victoria 10B St. S., Kitchener, ON N2G 1C5 Canada; 2 Department of Psychiatry & Behavioural Neurosciences, McMaster University, Hamilton, ON Canada; 3 Schlegel Research Institute for Aging, Waterloo, ON Canada; 4 School of Public Health and Health Systems, University of Waterloo, Waterloo, ON Canada; 5 Big Data and Geriatric Models of Care, McMaster University, Hamilton, ON Canada; 6 Department of Family Medicine, McMaster University, Hamilton, ON Canada; 7 Sinai Health System, Toronto, ON Canada; 8 Department of Health Research Methods, Evidence, and Impact, McMaster University, Hamilton, ON Canada

**Keywords:** Delirium, Nursing homes, Pain

## Abstract

**Background:**

Nursing home residents are frail, have multiple medical comorbidities, and are at high risk for delirium. Most of the existing evidence base on delirium is derived from studies in the acute in-patient population. We examine the association between clinical characteristics and medication use with the incidence of delirium during the nursing home stay.

**Methods:**

This is a retrospective cohort study of 1571 residents from 12 nursing homes operated by a single care provider in Ontario, Canada. Residents were over the age of 55 and admitted between February 2010 and December 2015 with no baseline delirium and a minimum stay of 180 days. Residents with moderate or worse cognitive impairment at baseline were excluded. The baseline and follow-up characteristics of residents were collected from the Resident Assessment Instrument-Minimal Data Set 2.0 completed at admission and repeated quarterly until death or discharge. Multivariate logistic regression was used to identify characteristics and medication use associated with the onset of delirium.

**Results:**

The incidence of delirium was 40.4% over the nursing home stay (mean LOS: 32 months). A diagnosis of dementia (OR: 2.54, *p* < .001), the presence of pain (OR: 1.64, p < .001), and the use of antipsychotics (OR: 1.87, p < .001) were significantly associated with the onset of delirium. Compared to residents who did not develop delirium, residents who developed a delirium had a greater increase in the use of antipsychotics and antidepressants over the nursing home stay.

**Conclusions:**

Dementia, the presence of pain, and the use of antipsychotics were associated with the onset of delirium. Pain monitoring and treatment may be important to decrease delirium in nursing homes. Future studies are necessary to examine the prescribing patterns in nursing homes and their association with delirium.

**Electronic supplementary material:**

The online version of this article (10.1186/s12877-018-0733-3) contains supplementary material, which is available to authorized users.

## Background

Delirium is a neuropsychiatric syndrome characterized by acute confusion and disturbance in attention that frequently complicates the hospital stays of older adults [1–3]. Its prevalence has been reported to range between 14% to 56% in acute care settings [4]. As a multifactorial syndrome, multiple predisposing (e.g., cognitive impairment and sensory impairments) and precipitating clinical factors (e.g. medications such as benzodiazepines and opioids, acute illnesses) have been previously identified [1, 2]. Delirium has also been found to be a negative prognosticator for survival [5].

Older adults who live in nursing home facilities are frail with multiple medical comorbidities, including dementia, that put them at higher risk for delirium [6]. A recent Canadian study showed that the prevalence of delirium is very low (< 0.5%) amongst older adults without dementia living in community and nursing home settings [5], but other studies have yielded a wide range of estimates, mainly point prevalence, from 1.4% to 70.3% among nursing home residents [4]. While studies suggest that some of the predisposing and precipitating factors for delirium in nursing homes (e.g. acute illness, depression, and dementia [7–9]) are similar to those found among hospitalized patients, there are notable differences in the course of delirium [2, 3] and patients’ exposure to stressors. For example, pain is highly prevalent among nursing homes residents [10]. The association between inadequate pain control and delirium has been studied repeatedly in the acute postoperative hip fracture population [11, 12], but there is little evidence from the nursing home setting. The use of acetaminophen for pain control has been shown to significantly reduce agitation among nursing home residents with moderate to severe dementia [13], but evidence suggests that analgesic use for long-term pain control has no impact on the rate of delirium [14].

The pathophysiology of delirium is not completely understood but both the cholingergic and dopaminergic pathways have been implicated [15]. Medications, such as antipsychotics and antidepressants, are known to affect these pathways and predispose elderly patients to delirium [1, 15]. In addition, polypharmacy is also a risk factor [8].

We sought to examine the association between clinical characteristics and medication use with the onset of delirium in nursing homes, with particular focus on the presence of pain and medication use. Our aim was to identify at-risk populations at the time of admission that could be considered in potential preventative interventions in nursing homes. Based on the available literature from inpatient settings, we hypothesized that dementia, pain, recent hospitalizations, and the use of antidepressants and antipsychotics would be associated with the onset of delirium.

## Methods

### Study design and setting

This was a retrospective cohort study using linked Resident Assessment Instrument-Minimal Data Set (RAI-MDS) 2.0 records from February 2010 to December 2015 from a large chain of for-profit nursing homes in Ontario, Canada. At the time of the study the nursing home chain consisted of 12 separate facilities serving approximately 1500 residents at any given time across Ontario. Ethics approval was granted through the Hamilton Integrated Research Ethics Board.

### Population

Nursing home residents who were over the age of 50, with two or more sequential RAI-MDS assessments (i.e. minimum stay of 180 days), and admitted to the nursing home between February 2010 and December 2015 were included in the cohort. Residents who had a Confusion Assessment Method (CAM) defined delirium at baseline were excluded. We excluded residents with moderate to severe cognitive impairment based on the Cognitive Performance Scale (CPS) [16] and those with moderate to severe communication difficulties at baseline given that some of the independent variables (e.g., pain) we examined are less reliable in this for these residents [17, 18]. CPS is a validated tool used to evaluate the level of cognitive impairment affecting a patient in a nursing home. On balance, we chose in favor of accuracy in our estimates rather than generalizability to all resident groups. Each nursing home resident was the unit of analysis.

### Data source

Census-level RAI-MDS 2.0 assessment records were used as the source of independent and dependent variables. The RAI-MDS 2.0 is a standardized interdisciplinary clinical assessment that examines over 300 items from 15 health domains to comprehensively describe resident characteristics, including socio-demographic variables, clinical characteristics, physical and cognitive status, medical diagnoses, major health problems and symptoms, current service use, and drug use [19–21]. The instrument has established psychometric properties [22–24], and has been used in many large scale studies [25–28]. It is implemented in Canadian nursing homes across seven provinces (Alberta, British Columbia, Manitoba, Newfoundland, Nova Scotia, Ontario, Saskatchewan), the Yukon, as well as internationally [29, 30]. In Canada, the RAI-MDS 2.0 is used to assess all residents at admission and every 3 months (quarterly) thereafter until death or discharge.

### Variables

Independent measures were selected a priori and included: demographic (age and sex), comorbidities (dementia, stroke, Parkinson’s disease, and depression), pain frequency, pain intensity, hospital admissions, functional status, and the use of medication classes (analgesics, anxiolytics, antipsychotics, and antidepressants). These measures in the RAI MDS 2.0 were examined at admission and at final assessment.

#### Pain evaluation

The presence of pain was defined in the RAI MDS 2.0 as any self-reported physical pain or discomfort occurring daily or less than daily over the 7 days prior to the assessment. Pain was characterized as mild, moderate, or horrible/excruciating. Persistent pain was defined as pain lasting intermittently recorded in at least two consecutive assessments). The RAI-MDS pain items have been validated in nursing home residents [23].

#### Functional status

Residents’ ability to perform Activities of Daily Living (ADL) and their continence status were used as surrogate markers for function. ADLs included dressing, eating, toilet use, personal hygiene, bathing, and locomotion. Each ADL item, except locomotion, was coded as independent if the resident required no assistance or only supervision. Locomotion was coded as independent if the resident could move between locations within one’s room and adjacent corridor on the same floor independently with or without supervision. If the resident used a wheelchair, locomotion was assessed once in chair. A score of 1 was given to each item that they were able to perform independently, as defined above. A cumulative score of 0 indicated complete ADL dependence while a score of 6 indicates the resident was able to perform all ADL items independently. Continence, both bowel and bladder, was measured and defined as residents having no more than one accident per week.

#### Medication classes

The classes of medications included were antidepressants, analgesics, antipsychotics, and anxiolytics. The number of days that each class of medications was used over the 7 days prior to the assessment was included. The specific medications included in each class was not available.

#### Hospital stays

At each RAI-MDS quarterly assessment, the number of times that a resident was admitted to the hospital was recorded. We included whether the resident was admitted to the hospital over the preceding quarter rather than the number of times.

#### Comorbidity status

The status of dementia, stroke, Parkinson’s disease, and depression were ascertained from RAI MDS 2.0.

### Dependent variable

The primary dependent measure was the first onset of delirium from baseline identified by sequential quarterly RAI-MDS 2.0 assessments. The endpoint (last assessment) was defined as the last available quarterly assessment before December 2015 among those without delirium, or the assessment recorded the first delirium event among those with delirium. Delirium was defined by the CAM diagnostic algorithm [31] using the six RAI-MDS delirium items [32], corresponding to the CAM items as follow: fluctuating course (mental fluctuations), inattention (easily distracted), disorganized thinking (episodes of disorganized speech; periods of altered perception), and altered level of consciousness (periods of restlessness; periods of lethargy). These items were counted in the delirium score if the time of onset was within seven days prior to the quarterly assessment to indicate an acute change as per CAM.

### Statistical analyses

The characteristics of the sample were summarized at admission (baseline) and last assessment (follow-up). The Chi-squared (**χ**^**2**^) test was used to compare categorical independent variables. McNemar’s test was used to compare change from baseline among categorical independent variables. The paired t-test was used to assess change from baseline among continuous variables. We used multivariate logistic regression to examine the association between our selected independent variables and incident delirium during the nursing home stay. Ad-hoc, we stratified the analyses according to a diagnoses of dementia at baseline and those who developed delirum. A *p*-value of ≤0.05 was considered to be significant. All analyses were performed using Statistical Analysis Software (SAS® 9.2, SAS Institute Inc., Cary, North Carolina). We report the study based on the STrengthening the Reporting of OBservational studies in Epidemiology (STROBE) statement [33].

## Results

### Sample description

A total of 3897 nursing home residents over the age of 55 were admitted to the nursing homes between February 2010 and December 2015 with two or more sequential RAI-MDS assessments (i.e. minimum stay of 180 days). Sequentially, we excluded 762 (19.1%) residents who had a CAM defined delirium at baseline, 1486 residents (37.3%) with moderate to severe cognitive impairment based on the CPS, and 78 residents (2.0%) with moderate to severe communication difficulties. One thousand five hundred seventy one residents were included in this study.

Overall, 40.4% (634) of residents had at least one delirium episode during their nursing home stay. Table 1 summarizes resident characteristics at baseline (admission assessment) and follow-up. Residents were of advanced age (83.3 ± 9.7) and predominantly female. A diagnosis of dementia, ADL impairment, reporting pain, bowel incontinence, and the use of analgesics and antidepressants were common at baseline. Analgesics and antidepressants were commonly prescribed, whereas antipsychotics and anxiolytics were less common at baseline. Residents that developed delirium during their nursing home stay had an average follow-up of 1.7 years (6.8 ± 5.1 quarterly assessments) compared to 2.2. years for those that did not develop delirium (8.9 ± 6.7 quarterly assessments). Overall, there was a trend toward increasing morbidity from baseline among all patients.

**Table 1:** Resident characteristics at baseline (admission) and follow-up for the sample (*N* = 1571)

	Baseline	Follow-up^a^	*P* value
Mean age (SD)	83.3 (9.7)	84.9 (9.5)	
Male	531 (33.8%)	531 (33.8%)	
Stroke	378 (24.1%)	408 (26.0%)	<.001
Parkinson’s disease	122 (7.8%)	130 (8.3%)	0.02
Dementia	669 (42.6%)	720 (45.9%)	<.001
Depression	339 (21.6%)	399 (25.4%)	<.001
Pain	664 (42.3%)	780 (49.7%)	<.001
Recent hospital stays	210 (13.4%)	180 (11.5%)	0.09
Function			
ADL score (SD)^b^	2.7 (1.8)	1.9 (1.7)	<.001
Locomotion (independence)	989 (66.4%)	750 (49.7%)	<.001
Dressing (independence)	471 (30.1%)	235 (15.0%)	<.001
Eating (independence)	1413 (90.1%)	1165 (74.2%)	<.001
Toileting (independence)	586 (37.5%)	344 (22.0%)	<.001
Hygiene (independence)	493 (31.5%)	285 (18.2%)	<.001
Bathing (independence)	201 (13.5%)	117 (7.9%)	<.001
Bladder continence (independence)	794 (68.2%)	489 (43.0%)	<.001
Bowel continence (independence)	1139 (72.7%)	857 (54.6%)	<.001
Medications			
Mean number of medications	11.2 (4.7)	11.8 (5.3)	<.001
Antidepressants	719 (45.8%)	821 (52.4%)	< 001
Antipsychotics	278 (17.7%)	296 (18.9%)	0.20
Anxiolytics	258 (16.4%)	250 (15.9%)	0.60
Analgesics	1049 (66.7%)	1167 (74.4%)	<.001
Any CAM defined delirium	0 (0.0%)	634 (40.4%)	<.001

*P*-value represents the results of McNemar’s test for paired binary variables and t-test for paired continuous variables

*ADL* activities of daily living, *N/A* not available

^a^The cohort was followed until delirium incidence, death, discharge, or the end of the study period

^b^*ADL* score was the sum of *ADL* items that each resident was able to perform. A score of 0 indicates complete *ADL* dependence while a score of 6 indicates the resident was able to perform all *ADL* items independently

Among all residents, there was a significant increase in the use of antidepressants (*p* < .001) and analgesics (p < .001), but not in antipsychotics (p = 0.20) or anxiolytics (*p* = 0.60) from baseline. Ad-hoc, we further stratified the information by delirium onset and the results are summarized in Table 2. Similar results were found except there was a significant increase in the use of antipsychotics among those who developed delirium. Similar results were observed in an ad hoc analysis stratified by dementia status at baseline, except that there was a significant increase in the use of antipsychotics among those with no dementia (2.6% *p* = 0.01, see Additional file 1: Table S1).

**Table 2:** Change in medication use from baseline stratified by delirium onset

Medications	Delirium onset (*N* = 634 / 40.4%)	No Delirium onset (*N* = 937 / 59.6%)
Antidepressants	8.0% (< 0.001)	5.6% (< 0.001)
Antipsychotics	5.5% (< 0.001)	−1.9% (0.05)
Anxiolytics	−0.9% (0.52)	−0.1% (0.92)
Analgesics	8.7% (< 0.001)	7.1% (< 0.001)
Mean follow-up in years^a^ (SD)	1.7 (1.3)	2.2 (1.7)

Comparison to baseline based on McNemar’s test for paired binary variables

^a^The cohort was followed until delirium incidence, death or discharge

### Association between resident characteristics and delirium onset

Table 3 summarizes the association between resident characteristics and delirium onset. The presence of dementia (OR: 2.54, *p* < .001), pain (OR: 1.64, p < .001), and the use of antipsychotics (OR: 1.88, *p* < .001) were significantly associated with the onset of delirium and had robust effect sizes. There were no significant associations between delirium and ADL impairment, depression, or other medication classes.

**Table 3:** Demographic and clinical characteristics associated with the onset of delirium

	OR	95% CI	*P* value
Age (SD)	1.00	0.98–1.01	0.57
Male	0.96	0.75–1.23	0.75
Stroke	1.03	0.79–1.34	0.82
Parkinson’s disease	0.89	0.58–1.37	0.60
Dementia	**2.54**	**1.99–3.25**	**<.001**
Depression	0.95	0.72–1.24	0.68
Pain	**1.64**	**1.25–2.16**	**<.001**
Persistent pain	1.01	0.77–1.32	0.96
Recent hospital stay	0.98	0.68–1.41	0.80
Function			
ADL score (SD)	0.98	0.90–1.06	0.61
Bowel incontinence	1.25	0.98–1.60	0.08
Medications			
Mean number of medications	0.99	0.97–1.02	0.59
Antidepressants	1.19	0.93–1.52	0.17
Antipsychotics	**1.88**	**1.40–2.51**	**<.001**
Anxiolytics	1.29	0.93–1.77	0.12
Analgesics	0.80	0.60–1.08	0.14

Results reaching statistical significance are bolded

*OR* = odds ratio; *CI* = confidence interval; *ADL* = activities of daily living

Ad hoc analyses examined the same associations stratified by dementia status.Among residents without dementia, the presence of pain (OR: 2.27, *p* < .001) and the use of antipsychotics (OR: 2.24, *p* < .001) were significantly associated with delirium. Among residents with dementia, the use of antipsychotics (OR: 1.65, *p* = 0.008) and an increase in ADL score (OR: 0.85, *p* = 0.007) were associated with delirium (see Additional file 2: Table S2).

## Discussion

We examined the association between clinical characteristics and medication use with the incidence of delirium during the nursing home stay. The study helps to establish the high incidence of delirium in nursing homes and it’s relationship to pain and antipsychotics. There have been smaller studies with similar objectives in subgroups of nursing home residents or outside of North America. At time of writing, to our knowledge this was the largest multi-site study among nursing home residents to be published.

The incidence of delirium was 40.4% over the residents’ nursing home stay, suggesting that delirium is not uncommon during the life course in nursing homes. Dementia is known to be a risk factor for delirium [34, 35], and it was strongly associated with delirium (OR: 2.54, *p* < .001) in our study. The incidence of delirium was 51.0% among those with dementia and 30.7% among those without dementia. Several studies have identified older age as an important risk factor for delirium in the acute setting [36–38]. This association was not observed in this study, perhaps due to the relative lack of variation in nursing homes.

The presence of pain was significantly associated with delirium overall (OR: 1.64, p < .001), and also among residents without dementia (OR: 2.27, *p* < .001). We did not find pain to be a significantly associated with delirium among residents with dementia, which may be due to poor detection as pain assessment is more challenging for those with even mild cognitive impairment (note: we excluded those with moderate to severe cognitive impairment for this reason). Pain is less likely to be a result of delirium, while it has been well established that pain predisposes one to delirium [1]. On the other hand, analgesic use might have been a confounder as the use of certain analgesics, such as opioid, predisposes one to delirium [1]. However, indications for medication use was not available nor in the scope of this sudy. Overall these findings suggest that pain is an important focus for the prevention of delirium in nursing homes. More vigilant monitoring of pain may decrease the frequency of delirium in nursing homes. More prospective studies on the safety of analgesics in nursing homes are required.

The use of antipsychotics was significantly associated with the onset of delirium. The increased use of antipsychotics among residents who developed delirium may be a result of trying to treat the hyperactive delirium with more agitated behaviours. However, antipsychotics may also be used to treat behavioural symptoms associated with various psychiatric illnesses. The use of both antidepressants and analgesics increased from baseline in residents with or without dementia at baseline. Although the type of analgesics and their indications were not available, this study provides information on the pattern of medication use among nursing home residents. Regular medication review would likely be beneficial as the original indications for some medications do not always persist. More research is needed to examine the appropriateness of prescribing patterns.

This study has several limitations. This was a retrospective cohort study and associations may be confounded by other unmeasured variables and no causative effect can be established. Quarterly assessments of pain, function, medication use, and delirium were assessed based on the 7 days prior to the assessment date. Therefore, changes in characteristics that were found to be associated with delirium were most likely present at the time of delirium. Given that our follow-up observation periods were based on the 7 days prior to the assessment date, we expect that we are underestimating the true incidence and frequency of delirium throughout the residents stay [3]. Also, delirium is thought to be under detected overall [39]. However, any underestimations would render our effect sizes as being conservative. We also did not perform cluster analyses to detect differences among between nursing homes because it was not our research question, and would not remove concern for external validity. Lastly, we did not have access to the raw medication administration records, which may confound the associations we found between different classes of medications and delirium. For instance, certain medications can be used as either an antidepressant or an anxiolytic. Future studies should examine subclasses of medications, such as serotonin reuptake inhibitors and benzodiazepines. We did not perform a Cox proportional hazard regression because our aims did not include time to event, and the follow-up observation periods were not ideal for ad-hoc analyses.

## Conclusions

This was a large study examining the factors associated with the onset of delirium over the nursing home stay. The results may be generalized to nursing homes across Canada as nursing home residents across Canada share similar clinical and epidemiologic characteristics [40]. Dementia, pain, and the use of antipsychotics were associated with the incidence of delirium. These findings point to the importance of pain detection, treatment, and monitoring in nursing homes as pain may contribute to delirium. Also, indications for analgesics and antidepressants to treat, such as pain, mood symptoms, and behavioral and psychological symptoms of dementia, should be carefully examined given the significant increase in their prescription. More research is needed to examine the appropriateness of prescribing patterns in nursing homes.

## Additional files

Additional file 1: Table S1. Change in medication use from baseline stratified by dementia status at baseline. This is a table summarizing the the change in the use of different medication classes described in text from baseline stratified by dementia status at baseline. (DOCX 14 kb)

Additional file 2: Table S2. Demographic and clinical characteristics associated with the onset of delirium stratified by dementia status. The is a table summarizing the logistic regression results looking at factors associated with the onset of delirium. The study population was divided into two groups based on the presence or absence of dementia at the time of delirium onset or their last available assessment in those without delirium. (DOCX 16 kb)

## Abbreviations

ADL: Activities of daily living
CAM: Confusion Assessment Method
CPS: Cognitive performance scale
RAI-MDS: Resident Assessment Instrument-Minimal Data Set
STROBE: STrengthening the reporting of observational studies in epidemiology
