# Supplementary material for: Clinical characteristics associated with the onset of delirium among long-term nursing home residents
Source: BMC Geriatr. 2018 Feb 2;18:39. doi: 10.1186/s12877-018-0733-3 (PMC5797375; doi:10.1186/s12877-018-0733-3)
Supplement: Supplementary file 1 — Change in medication use from baseline stratified by dementia status at baseline. This is a table summarizing the the change in the use of different medication classes described in text from baseline stratified by dementia status at baseline. (DOCX 14 kb) [file 12877_2018_733_MOESM1_ESM.docx]

Table S1: Change in medication use from baseline stratified by dementia status at baseline

| **Medications** | **Dementia (N=718)** | **No Dementia (N=848)** |
| --- | --- | --- |
| Antidepressants | 7.2% (<0.001) | 6.0% (<0.001) |
| Antipsychotics | -0.7% (0.63) | 2.6% (0.01) |
| Anxiolytics | -1.3% (0.26) | 0.2% (0.85) |
| Analgesics | 8.5% (<0.001) | 7.1% (<0.001) |
| Mean follow-up in years * (SD) | 1.8 (1.3) | 2.2 (1.7) |

Comparison to baseline based on McNemar’s test for paired binary variables.

*The cohort was followed until delirium incidence, death or discharge.
