# Supplementary material for: Clinical characteristics associated with the onset of delirium among long-term nursing home residents
Source: BMC Geriatr. 2018 Feb 2;18:39. doi: 10.1186/s12877-018-0733-3 (PMC5797375; doi:10.1186/s12877-018-0733-3)
Supplement: Supplementary file 2 — Demographic and clinical characteristics associated with the onset of delirium stratified by dementia status. The is a table summarizing the logistic regression results looking at factors associated with the onset of delirium. The study population was divided into two groups based on the presence or absence of dementia at the time of delirium onset or their last available assessment in those without delirium. (DOCX 16 kb) [file 12877_2018_733_MOESM2_ESM.docx]

**Table S2:** Demographic and clinical characteristics associated with the onset of delirium stratified by dementia status.

|  | **No Dementia**  **N=731** | | | **Dementia**  **N=629** | | |
| --- | --- | --- | --- | --- | --- | --- |
|  | **OR** | **95% CI** | **P value** | **OR** | **95% CI** | **P value** |
| Overall |  |  |  | | |  |
| Age (SD) | 0.99 | 0.98 – 1.01 | 0.28 | 1.00 | 0.98 – 1.02 | 0.67 |
| Male | 0.84 | 0.58 – 1.22 | 0.37 | 0.99 | 0.68 – 1.36 | 0.94 |
| Stroke | 0.98 | 0.67 – 1.42 | 0.90 | 1.09 | 0.74 – 1.62 | 0.66 |
| Parkinson’s disease | 1.21 | 0.66 – 2.22 | 0.54 | 0.71 | 0.39 – 1.29 | 0.26 |
| Depression | 1.13 | 0.76 – 1.69 | 0.54 | 0.81 | 0.55 – 1.19 | 0.28 |
| Pain | **2.27** | 1.50 – 3.43 | **<0.001** | 1.25 | 0.86 – 1.83 | 0.25 |
| Persistent pain | 1.03 | 0.71 – 1.51 | 0.86 | 0.92 | 0.62 – 1.39 | 0.70 |
| Recent hospital stays | 1.31 | 0.81 – 2.12 | 0.26 | 0.73 | 0.40 – 1.35 | 0.33 |
| Function |  | | | | |  |
| ADL score (SD)* | 1.13 | 1.00 – 1.27 | 0.053 | **0.85** | 0.75 – 0.95 | **0.007** |
| Bowel incontinence | 1.36 | 0.95 – 1.95 | 0.09 | 1.12 | 0.79 – 1.59 | 0.53 |
| Medications |  | | | | |  |
| Number of medications | 0.99 | 0.96 – 1.02 | 0.41 | 1.00 | 0.96 – 1.05 | 0.91 |
| Antidepressants | 1.42 | 0.99 – 2.06 | 0.06 | 0.97 | 0.69 – 1.37 | 0.85 |
| Antipsychotics | **2.24** | 1.39 – 3.61 | **<0.001** | **1.65** | 1.14 – 2.39 | **0.008** |
| Anxiolytics | 1.40 | 0.92 – 2.11 | 0.11 | 1.12 | 0.66 – 1.90 | 0.68 |
| analgesics | 0.94 | 0.59 – 1.50 | 0.79 | 0.78 | 0.52 – 1.15 | 0.21 |

*ADL score was the sum of ADL items that each resident was able to perform. A score of 0 indicates complete ADL dependence while a score of 6 indicates the resident was able to perform all ADL items independently
OR = odds ratio; CI = confidence interval; ADL = activities of daily living
